# The Diseases of Children

**Published:** 1884-08

**Authors:** 


**The Diseases of Children.** A Handbook for Practitioners and Students. By ARMAND SEMPLE, M. R. C. P., London, Physician Northeastern Hospital for Children, Etc., London. G. P. Putnam's Sons, 27 and 29 West 23d Street, New York. 1884.

The economy of nature prescribes babies as its most condant and abundant blessing, and as long as there are babies the tiseases of childhood will occupy a large share of the attention of medical men. Perhaps no class of diseases are more frequently encountered by the young medical man, and a book like the one before us he will find of the greatest value. While not a large book it yet covers the ground indicated by its title. The style is at once clear, interesting and concise. For the student we consider it the best book on its special subjects published.

